# Supplementary material for: Interplay of biotic and abiotic factors shapes tree seedling growth and root-associated microbial communities
Source: Commun Biol. 2024 Mar 22;7:360. doi: 10.1038/s42003-024-06042-7 (PMC10960049; doi:10.1038/s42003-024-06042-7)
Supplement: Supplementary file 2 — Supplementary Material [file 42003_2024_6042_MOESM2_ESM.pdf]

**SUPPLEMENTARY INFORMATION**

# Interplay of biotic and abiotic factors shapes tree seedling growth and root-associated microbial communities

Joey Chamard[123+], Maria Faticov[123*+], F. Guillaume Blanchet[1,4,5], Pierre-Luc Chagnon[6,7], and Isabelle Laforest-Lapointe[123*]

[1]Département de biologie, Université de Sherbrooke, Sherbrooke, Québec, Canada

[2]Centre Sève, Département de Biologie, Université de Sherbrooke, Sherbrooke, Québec, Canada

[3]Centre d'Étude de la Forêt Université du Québec à Montréal, Montréal, Québec, Canada

[4]Département de mathématiques, Université de Sherbrooke, Sherbrooke, Québec, Canada

[5]Département des sciences de la santé communautaire, Université de Sherbrooke, Sherbrooke, Québec, Canada

[6]Agriculture and Agri-food Canada, Saint-Jean-sur-Richelieu, Québec, Canada

[7]Département des Sciences Biologiques, Université de Montréal, Montréal, Québec, Canada

[+]These authors contributed equally.

[*]Co-corresponding authors: isabelle.laforest.lapointe@gmail.com, maria.faticov@gmail.com

## SUPPLEMENTARY TABLES AND FIGURES

**Supplementary Table 1.** Site specific differences between Sutton and Mégantic. Comparisons were modelled using non-parametric Kruskal-Wallis tests. Bold lines indicate statistical significance ($p \leq 0.05$).

| **Parameters** | **Sutton** (mean ±SD) | | **Mégantic** (mean ±SD) | |
|---|---|---|---|---|
| *Site* | | | | |
| Number of seedlings | *n* = 51 | | *n* = 47 | |
| **Gradient elevation (m.a.s.l.)** | **649.37 (567-725)** | *** | **736.45 (675-796)** | ↑ |
| **Canopy openness (%)** | **12.59 ± 5.08** | * | **14.54 ± 5.19** | ↑ |
| Distance to flowbed (m) | 32.50 ± 20.98 | | 25.70 ± 18.69 | |
| *Soil* | | | | |
| pH | 4.56 ± 0.41 | | 4.56 ± 0.50 | |
| **Moisture (g/g)** | **2.08 ± 1.33** | * | **1.61 ± 1.19** | ↓ |
| Total C (g/Kg) | 232.18 ± 120.30 | $^m$ | 191.47 ± 109.14 | |
| Total N (g/Kg) | 14.30 ± 6.62 | | 11.99 ± 5.27 | |
| Phosphorus (mg/Kg) | 35.08 ± 37.41 | $^m$ | 51.89 ± 51.75 | |
| **Calcium (mg/Kg)** | **752.29 ± 851.91** | *** | **1 682.09 ± 1 548.87** | ↑ |
| **Magnesium (mg/Kg)** | **97.65 ± 65.87** | *** | **204.43 ± 166.72** | ↑ |
| Potassium (mg/Kg) | 189.43 ± 99.79 | | 193.45 ± 90.32 | |
| *Seedling* | | | | |
| Age (years) | 13.49 ± 3.84 | | 12.94 ± 4.86 | |
| **Annual growth (mm)** | **30.71 ± 19.92** | *** | **45.96 ± 28.32** | ↑ |
| Height (cm) | 49.88 ± 20.43 | | 43.92 ± 11.33 | |
| Neck diameter | 7.48 ± 2.34 | $^m$ | 6.79 ± 2.13 | |
| **Foliar phosphorus (ppm)** | **1 305.57 ± 215.54** | ** | **1 464.82 ± 240.62** | ↑ |
| Foliar calcium (ppm) | 6 903.74 ± 2354.12 | | 6 343.84 ± 2 584.17 | |
| Foliar magnesium (ppm) | 1 704.45 ± 487.36 | $^m$ | 1 912.80 ± 501.34 | |
| **Foliar potassium (ppm)** | **6193.06 ± 1 389.97** | *** | **7 282.95 ± 1 384.93** | ↑ |
| *AMF colonisation* | | | | |
| **Root length colonisation (%)** | **52.96 ± 18.82** | * | **44.36 ± 14.09** | ↓ |
| Arbuscules (%) | 31.41 ± 14.36 | | 27.77 ± 11.24 | |
| Vesicles (%) | 11.04 ± 8.43 | | 10.6 ± 7.10 | |
| *DSE colonisation* | | | | |
| **Total colonisation (%)** | **37.49 ± 20.68** | *** | **22.89 ± 21.91** | ↓ |

Significance codes: $^m$ $p \leq 0.1$ * ≤ 0.05, ** ≤ 0.01, *** ≤ 0.001
Arrows on the right show if Mégantic's values were higher or lower than Sutton's.

**Supplementary Table 2**. Matrix correlations. (A and C) Multivariate correlations between environmental predictors and root microbial communities in Sutton and Mégantic, respectively. (B and D) Multivariate correlations between each pair of matrices of root microbial communities (i.e., fungal, bacterial, soil and root AMF). Shown are RV coefficients (multivariate generalizations of the squared correlation coefficient) and *p*-values. To remove redundancy, only the lower triangular portion of the microbial matrices were presented. Significant effects ($p \leq 0.05$) are shown in bold.

| **Sutton** | **AMF roots** | | **AMF soil** | | **Fungi** | | **Bacteria** | |
|---|---|---|---|---|---|---|---|---|
| **A. Environment** | **RV (%)** | ***p*-value** | **RV (%)** | ***p*-value** | **RV (%)** | ***p*-value** | **RV (%)** | ***p*-value** |
| Elevation and canopy openness | 13.5 | **0.025** | 17.3 | **0.025** | 24.7 | **< 0.001** | 22.6 | **< 0.001** |
| Host characteristics | 11.2 | 0.243 | 17.1 | 0.140 | 22.3 | **< 0.001** | 27.2 | **< 0.001** |
| Neighbouring plant community | 26.2 | 0.175 | 21.8 | 0.226 | 21.9 | 0.106 | 35.4 | 0.109 |
| Soil chemistry | 16.3 | **0.005** | 15.4 | **0.023** | 23 | **< 0.001** | 33.8 | **< 0.001** |
| Distance to conspecific, diameter to conspecific and AMF metrics | 21.7 | **0.002** | 9.7 | **0.016** | 23.2 | **< 0.001** | 22.9 | **0.006** |
| **B. Microbial matrices** | **RV (%)** | ***p*-value** | **RV (%)** | ***p*-value** | **RV (%)** | ***p*-value** | **RV (%)** | ***p*-value** |
| AMF roots | | | | | | | | |
| AMF soil | 56.7 | **< 0.001** | | | | | | |
| Fungi | 62.1 | **< 0.001** | 61.7 | **< 0.001** | | | | |
| Bacteria | 50.4 | **< 0.001** | 50.4 | 0.72 | 82.3 | **< 0.001** | | |
| **Mégantic** | **AMF roots** | | **AMF soil** | | **Fungi** | | **Bacteria** | |
| **C. Environment** | **RV (%)** | ***p*-value** | **RV (%)** | ***p*-value** | **RV (%)** | ***p*-value** | **RV (%)** | ***p*-value** |
| Elevation and canopy openness | 14 | **0.014** | 11.7 | 0.156 | 19.5 | **0.001** | 44.6 | **< 0.001** |
| Host characteristics | 6.6 | 0.183 | 23.9 | **0.003** | 34.1 | **< 0.001** | 17.9 | **0.009** |
| Neighbouring plant community | 13.9 | 0.426 | 32 | **0.005** | 41.7 | **< 0.001** | 38.6 | **< 0.001** |
| Soil chemistry | 3.2 | 0.433 | 11.7 | 0.158 | 19.7 | **< 0.001** | 26.7 | **< 0.001** |
| Distance to conspecific, diameter to conspecific and AMF metrics | 8.6 | **0.001** | 18.4 | 0.144 | 27.8 | **< 0.001** | 26.2 | **< 0.001** |
| **D. Microbial matrices** | **RV (%)** | ***p*-value** | **RV (%)** | ***p*-value** | **RV (%)** | ***p*-value** | **RV (%)** | ***p*-value** |
| AMF roots | | | | | | | | |
| AMF soil | 23.2 | **0.013** | | | | | | |
| Fungi | 29.3 | **< 0.001** | 67.8 | **< 0.001** | | | | |
| Bacteria | 26.2 | **0.007** | 61.9 | **< 0.001** | 86.3 | **< 0.001** | | |

**Supplementary Table 3.** The change in relative abundance of fungal functional guilds along seedling annual growth in Sutton (A) and Megantic (B). Shown are t-values, *P*-values, standardized regression coefficients and its associated standard errors (SE) for the linear models.

| **A. Sutton** | **t-value** | ***P*-value** | **Coef** | **SE (Coef)** |
|---|---|---|---|---|
| Mutualists | -0.09 | 0.933 | -1.57 | 15.56 |
| Saprotrophs | 0.98 | 0.332 | 13.47 | 13.76 |
| Pathogens | 0.62 | 0.538 | 3.04 | 4.90 |
| Other | -0.70 | 0.487 | -7.54 | 2.79 |
| Unknown | -0.71 | 0.484 | -8.19 | 11.60 |
| **B. Megantic** | **t-value** | ***P*-value** | **Coef** | **SE (Coef)** |
| Mutualists | 0.17 | 0.865 | 2.86 | 0.26 |
| Saprotrophs | -0.24 | 0.808 | -5.31 | 21.76 |
| Pathogens | 1.34 | 0.187 | 0.15 | 0.11 |
| Other | -1.32 | 0.192 | -0.28 | 0.09 |
| Unknown | -0.19 | 0.847 | -4.36 | 22.48 |

**Supplementary Table 4**. Differential abundance analysis on fungal families and genus from multivariate generalized linear models for Sutton (A, C) and Mégantic (B, D). Arrows on the right shows whether the relative abundance of fungal families and genus was significantly higher or lower with higher seedling growth. Significant effects ($p \leq 0.05$) are shown in bold.

| **Sutton** | | | | **Mégantic** | | |
|---|---|---|---|---|---|---|
| **A. Fungal family** | **Dev** | ***P*-value** | | **B. Fungal family** | **Dev** | **P-value** |
| *Archaeorhizomycetaceae* | 2.595 | 0.571 | | *Archaeorhizomycetaceae* | 3.607 | 0.536 |
| *Herpotrichiellaceae* | 2.912 | 0.540 | | *Melanommataceae* | 0.971 | 0.937 |
| ***Dermateaceae*** | **9.691** | **0.024** | ↓ | *Herpotrichiellaceae* | 0.55 | 0.965 |
| *Helotiaceae* | 3.158 | 0.508 | | *Dermateaceae* | 1.383 | 0.902 |
| *Hyaloscyphaceae* | 3.264 | 0.508 | | *Helotiaceae* | 0.034 | 0.997 |
| *Leotiaceae* | 1.156 | 0.918 | | *Hyaloscyphaceae* | 1.369 | 0.902 |
| *Vibrisseaceae* | 0 | 0.989 | | *Leotiaceae* | 2.197 | 0.824 |
| ***Chaetosphaeriaceae*** | **8.804** | **0.043** | ↓ | *Chaetosphaeriaceae* | 0.008 | 0.997 |
| *Cephalothecaceae* | 0.029 | 0.989 | | *Hypocreaceae* | 2.223 | 0.824 |
| *Strophariaceae* | 0.76 | 0.947 | | *Nectriaceae* | 7.011 | 0.098 |
| *Tricholomataceae* | 2.328 | 0.643 | | *Clavariaceae* | 0.772 | 0.955 |
| *Auriculariales_fam_Incertae_sedis* | 0.203 | 0.989 | | *Entolomataceae* | 0.468 | 0.965 |
| *Clavulinaceae* | 2.734 | 0.989 | | *Hygrophoraceae* | 0.495 | 0.965 |
| *Russulaceae* | 4.027 | 0.372 | | *Strophariaceae* | 0.676 | 0.955 |
| *Sebacinaceae* | 0.287 | 0.989 | | *Tricholomataceae* | 0.002 | 0.997 |
| *Thelephoraceae* | 1.123 | 0.918 | | *Russulaceae* | 0.391 | 0.965 |
| *Hydnodontaceae* | 0.287 | 0.989 | | *Hydnodontaceae* | 1.133 | 0.916 |
| *Glomeraceae* | 4.71 | 0.275 | | *Glomeraceae* | 2.298 | 0.824 |
| *Mortierellaceae* | 0.441 | 0.976 | | *Mortierellaceae* | 1.974 | 0.824 |
| *Unknown* | 0.196 | 0.989 | | *Unknown* | 2.27 | 0.824 |

| **Sutton** | | | | **Mégantic** | | |
|---|---|---|---|---|---|---|
| **C. Fungal genus** | **Dev** | ***P*-value** | | **D. Fungal genus** | **Dev** | **P-value** |
| *Archaeorhizomyces* | 2.595 | 0.655 | | *Archaeorhizomyces* | 3.607 | 0.467 |
| *Cladophialophora* | 1.517 | 0.871 | | *Cladophialophora* | 0.178 | 0.998 |
| ***Pezicula*** | **9.023** | **0.024** | ↓ | *Pezicula* | 0.717 | 0.974 |
| *Hymenoscyphus* | 1.950 | 0.769 | | *Gyoerffyella* | 1.732 | 0.836 |
| ***Gyoerffyella*** | **10.478** | **0.018** | ↑ | *Hyaloscypha* | 1.702 | 0.836 |
| *Hyaloscypha* | 3.049 | 0.562 | | *Lachnum* | 1.258 | 0.914 |
| *Gorgomyces* | 0.03 | 0.995 | | *Trichoderma* | 3.055 | 0.591 |
| *Phialocephala* | 0.363 | 0.987 | | *Ilyonectria* | 2.241 | 0.760 |
| *Vibrissea* | 0.041 | 0.995 | | *Cephalotheca* | 0 | 1.000 |
| *Cephalotheca* | 0.029 | 0.995 | | *Cyclocybe* | 5.915 | 0.142 |
| *Galerina* | 0.076 | 0.970 | | *Clavulinopsis* | 0.124 | 0.998 |
| *Mycena* | 0.295 | 0.705 | | *Entoloma* | 0.644 | 0.974 |
| *Renatobasidium* | 0.203 | 0.987 | | *Hygrocybe* | 0.176 | 0.998 |
| *Clavulina* | 2.403 | 0.697 | | *Galerina* | 0.836 | 0.964 |
| *Lactarius* | 3.258 | 0.532 | | *Mycena* | 0.034 | 1.000 |
| *Russula* | 1.053 | 0.945 | | *Renatobasidium* | 0.011 | 1.000 |
| *Sebacina* | 0.287 | 0.987 | | *Lactarius* | 0.011 | 1.000 |
| *Tomentella* | 0.507 | 0.987 | | *Russula* | 0.621 | 0974 |
| *Mortierella* | 0.220 | 0.987 | | *Mortierella* | 1.789 | 0.836 |
| *Unknown* | 0.462 | 0.987 | | *Unknown* | 1.551 | 0.849 |

**Supplementary Table 5.** Primer pairs and polymerase chain reaction (PCR) conditions for all microbial groups.

| PCR | Bacteria | Fungi | Arbuscular mycorrhizae | |
|---|---|---|---|---|
| **Primer pairs** | 799F: AACMGGATTAGATACCCK<br>1115R: AGGGTTGCGCTCGTTG | ITS-1F: CTTGGTCATTTAGAGGAAGTAA<br>ITS2: GCTGCGTTCTTCATCGATGC | AML2: GAACCCAAACACTTTGGTTTCC<br>WANDA: CAGCCGCGGTAATTCCAGCT | |
| **Primer** | V5-V6-V7 region[1] | ITS1 region[2,3] | V4-V5 region[4] | |
| **Approach** | One-step PCR | One-step PCR | Two-steps PCR | |
| **Controls** | + & - | - | - | |
| **PCR** | | | **Step 1** | **Step 2** |
| ***Denaturation*** | 98ºC; 30 sec. | 98ºC; 30 sec. | 98ºC; 30 sec. | 98ºC; 30 sec. |
| ***Cycles*** | $n = 33$ | $n = 33$ | $n = 31$ | $n = 15$ |
| ***Amplification conditions*** | 98ºC; 15 sec.<br>64ºC; 30 sec. | 98ºC; 15 sec.<br>55ºC; 30 sec. | 98ºC; 15 sec.<br>55ºC; 30 sec. | 98ºC; 15 sec.<br>60ºC; 30 sec. |
| ***Elongation*** | 72ºC; 10 min | 72ºC; 10 min | 72ºC; 10 min | 72ºC; 10 min |
| **Reagents** | | | | |
| ***5X Phusion HF Buffer*** | 1X | 1X | 1X | 1X |
| ***dNTPs (10mM)*** | 0.2 mM | 0.2 mM | 0.2 mM | 0.2 mM |
| ***DMSO*** | 3% | 3% | 3% | 3% |
| ***DNA template*** | 1 µl | 1 µl | 1 µl (1/10) | 1 µl (1/50) |
| ***Forward primer*** | 0.2 µM | 0.2 µM | 0.4 µM | 0.2 µM (barcoded) |
| ***Reverse primer*** | 0.2 µM | 0.2 µM | 0.4 µM | 0.2 µM (barcoded) |
| ***Phusion Hot Start II*** | 0.02 U/µl | 0.02 U/µl | 0.02 U/µl | 0.02 U/µl |

**Supplementary Table 6.** Summary statistics for each microbial dataset across the 98 samples.

| **Datasets*** | **Total sequences** | **Total ASVs** | **Sequence length (bp)** | **Sequences per sample** | | **ASVs per sample** | | **ASV sample occurrence** | |
|---|---|---|---|---|---|---|---|---|---|
| | | | Mean | Mean (range) | SD | Mean (range) | SD | Mean (range) | SD |
| Bacteria | 2 356 945 | 7 066 | *c*.300 | 23 010 (9 511-41 571) | 6 607 | 456 (240-675) | 101 | 6 1-98 | 11 |
| Fungi | 1 745 695 | 1 797 | *c*.250 | 17 813 (4 235-47 897) | 8 463 | 84 (47-130) | 18 | 5 1-82 | 8 |
| AMF roots | 344 267 | 182 | *c*.400 | 3 513 (1 616-3 946) | 412 | 19 (4-39) | 8 | 4 1-97 | 8 |
| AMF soil | 95 011 | 173 | *c*.400 | 970 (55-2 942) | 742 | 14 (3-29) | 6 | 8 1-66 | 10 |

*After filtering for quality, chimeras, and removing ASVs with less than 10 sequences.

**Supplementary Table 7.** Taxonomical annotation across microbial datasets.

| Datasets | Domain | Phylum | Class | Order | Family | Genera | Species |
|---|---|---|---|---|---|---|---|
| Bacteria | 100% | 99.9% | 99.4% | 95.4% | 81.1% | 63.2% | 3.5% |
| Fungi | 100% | 98.3% | 73.3% | 69.8% | 65.8% | 52.8% | 23.4% |
| Mycorrhizae | 100% | 89.7% | 89.7% | 38.3% | 37.7% | 18.3% | 2.3% |

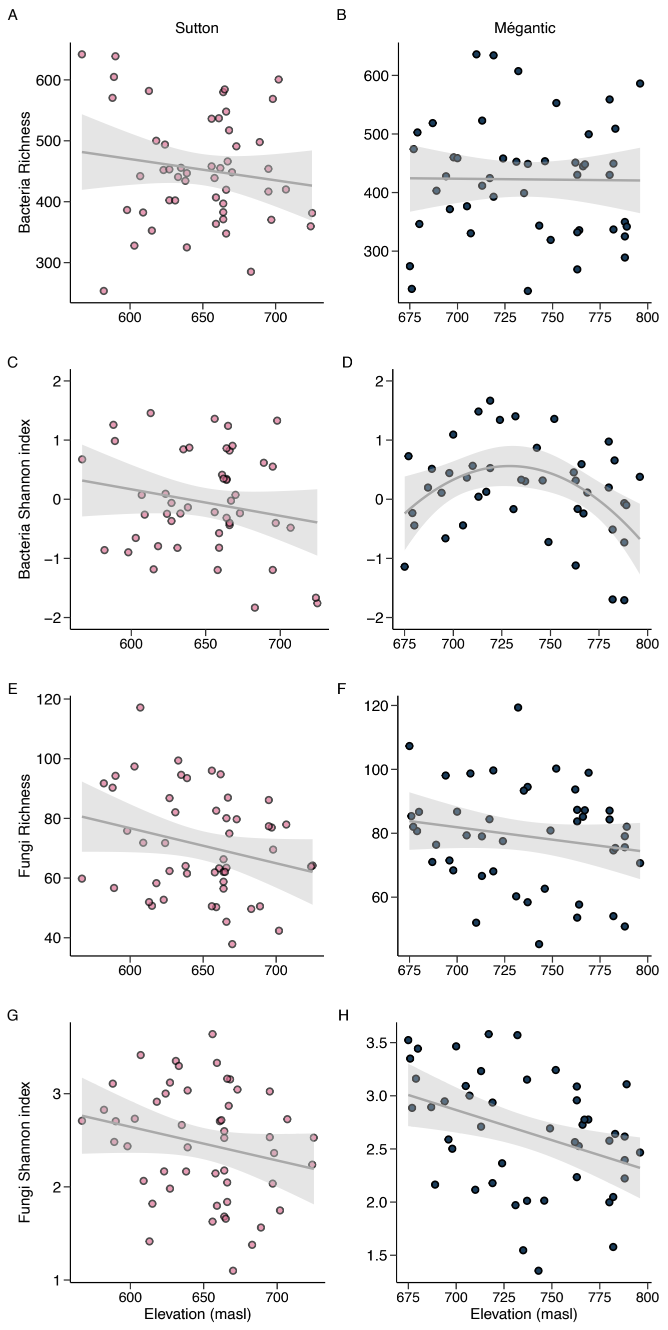


**Supplementary Figure 1.** Root microbial alpha-diversity across elevation at Sutton (A, C, E, G) and Mégantic (B, D, F, H). Grey line shading represents 95% estimation confidence interval of smoothed conditional means with a (A-C, E-G) linear regressions and (D) a quadratic regression. Panels C-D show normalized Shannon index. Panel D shows a significant quadratic relationship (p=0.006).

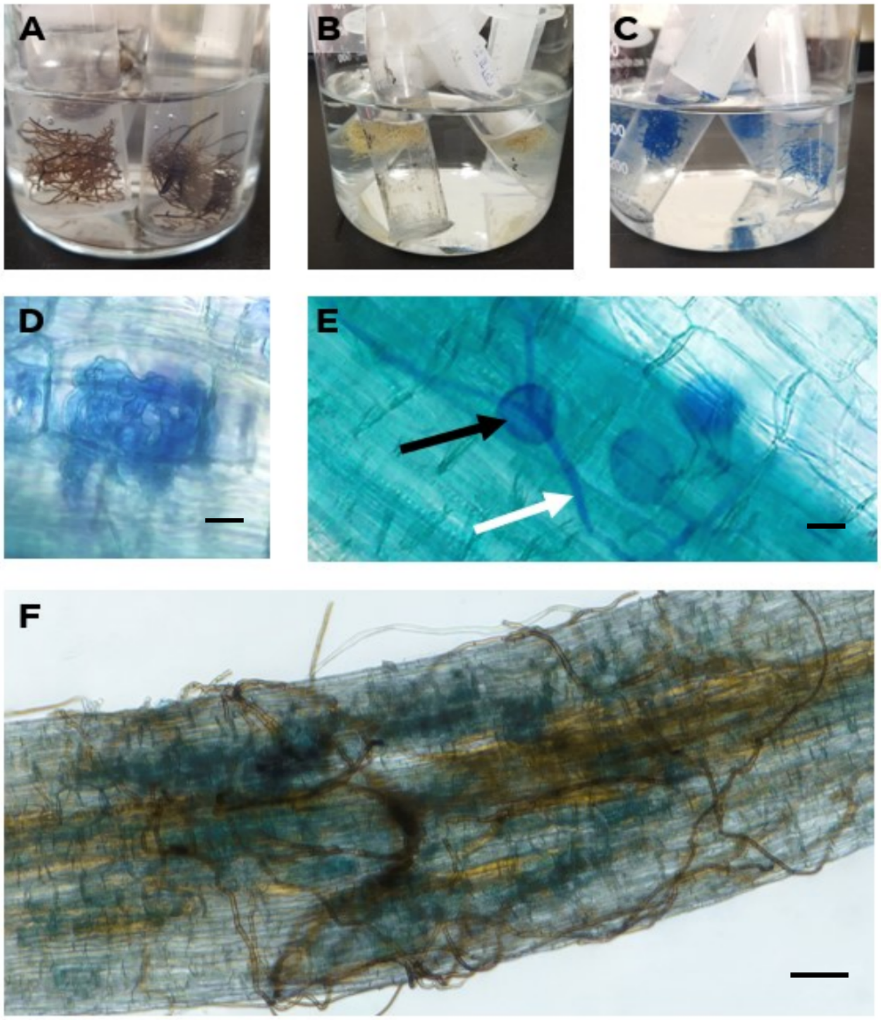


**Supplementary Figure 2.** Preparation of root coloration for visualization and quantification of fungal structures by light microscopy.

Panel (A) shows root samples before discoloration, (B) after discoloration, and (C) after coloration. Roots were mounted on slides for observation of arbuscular mycorrhizal structures following McGonnigle's (1990) protocol. Quantification of (D) arbuscules, (E) vesicles (black arrow) and hyphae (white arrow) as well as (F) dark septed endophyte (*brown* structures) colonisation was performed for all seedlings. Panel (F) shows co-occurrence of arbuscular mycorrhizal fungi (*blue*) and dark septed endophytes (*brown*) within a root section. Scale bars: D-E=20µm and F=100µm.

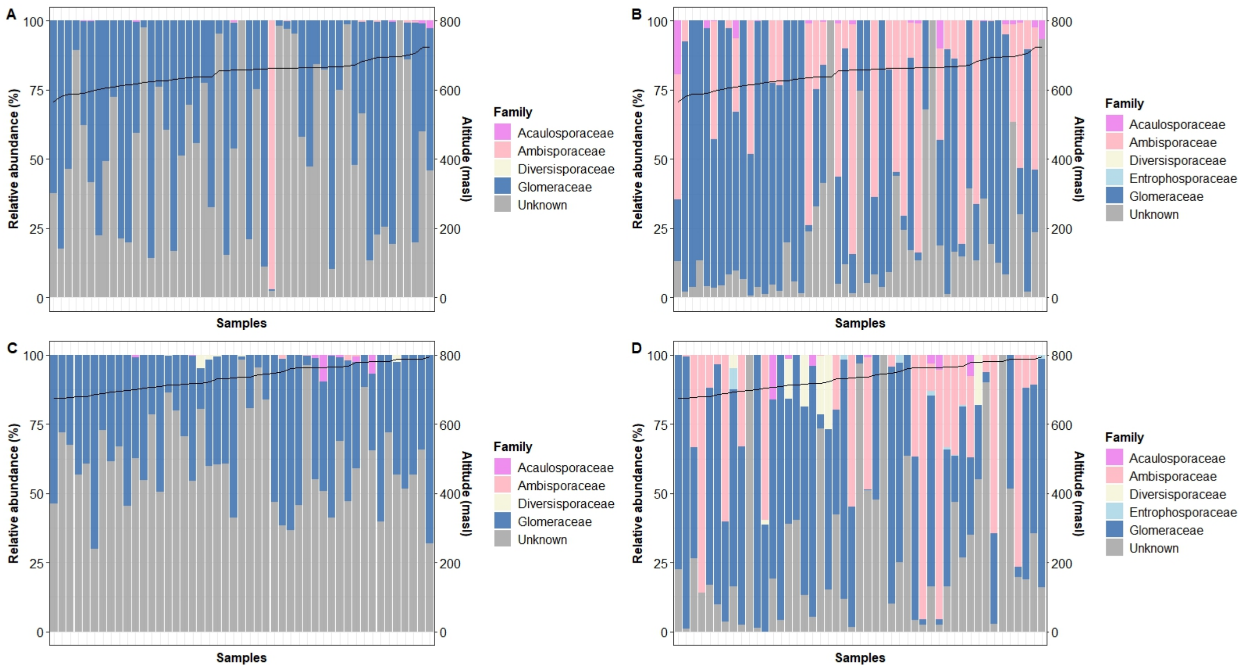


**Supplementary Figure 3.** Relative abundance of microbial families along two small-scale elevational gradients. (A and C) Relative abundance of arbuscular mycorrhizal fungi in roots. (B and D) Relative abundance of arbuscular mycorrhizal fungi in surrounding soil samples. Seedlings at Sutton (A, B) and Mégantic (C, D) are ordered by elevation. The right axis shows the elevational gradient, represented by the dark line on the graph, while the left axis illustrates the relative abundance of microbial families in each sample.

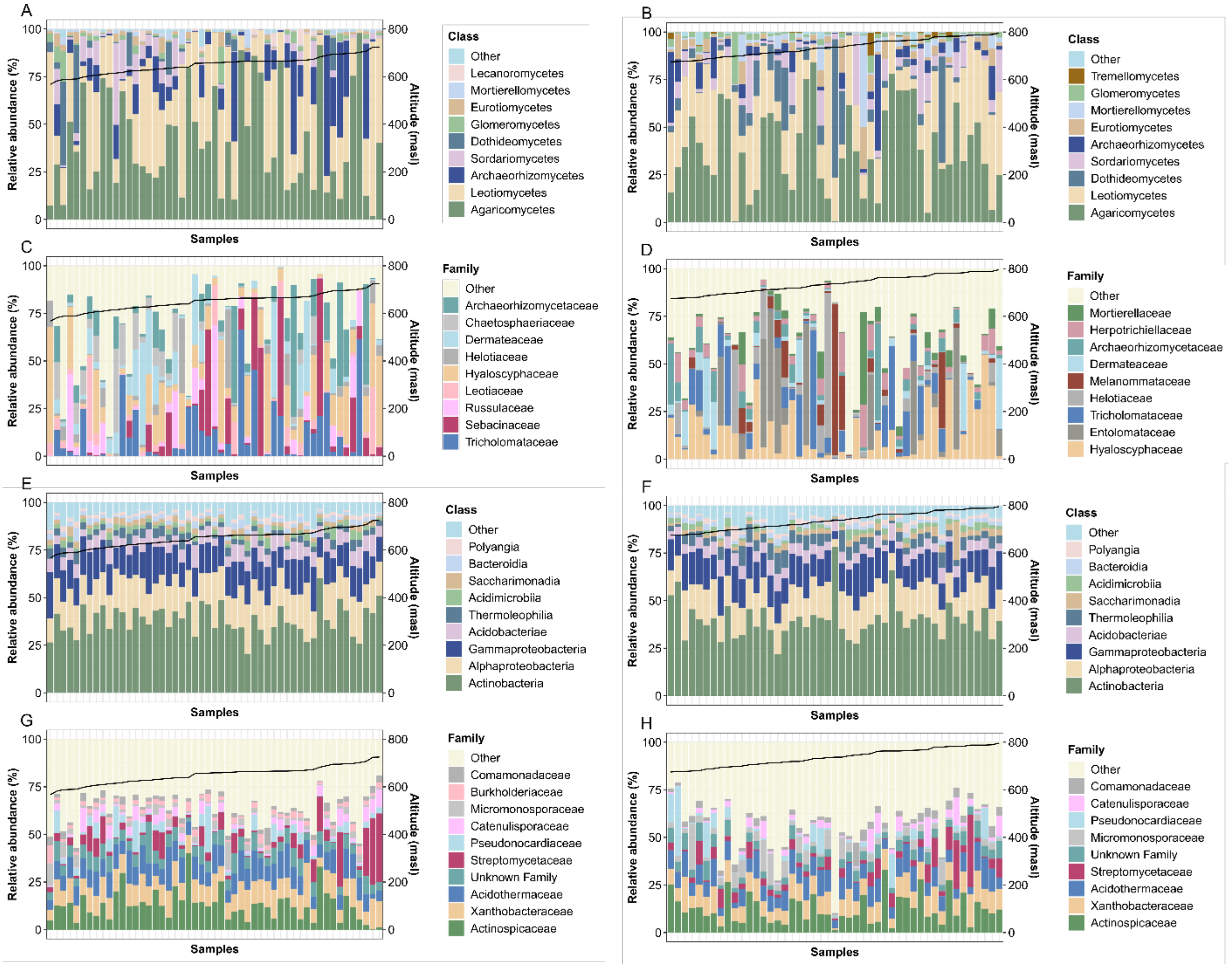


**Supplementary Figure 4**. Microbial relative abundance at the class and family levels along two elevational gradients. (A-D) Relative abundance of root fungi at the class (A-B) and family (C-D) levels. (E-H) Relative abundance of root bacteria at the class (E-F) and family (G-H) levels. Seedlings at Sutton (A, C, E, G) and Mégantic (B, D, F, H) are ordered by elevation. The right axis shows the elevational gradient, represented by the dark line, while the left axis shows the relative abundance of microbial classes and families in each sample.

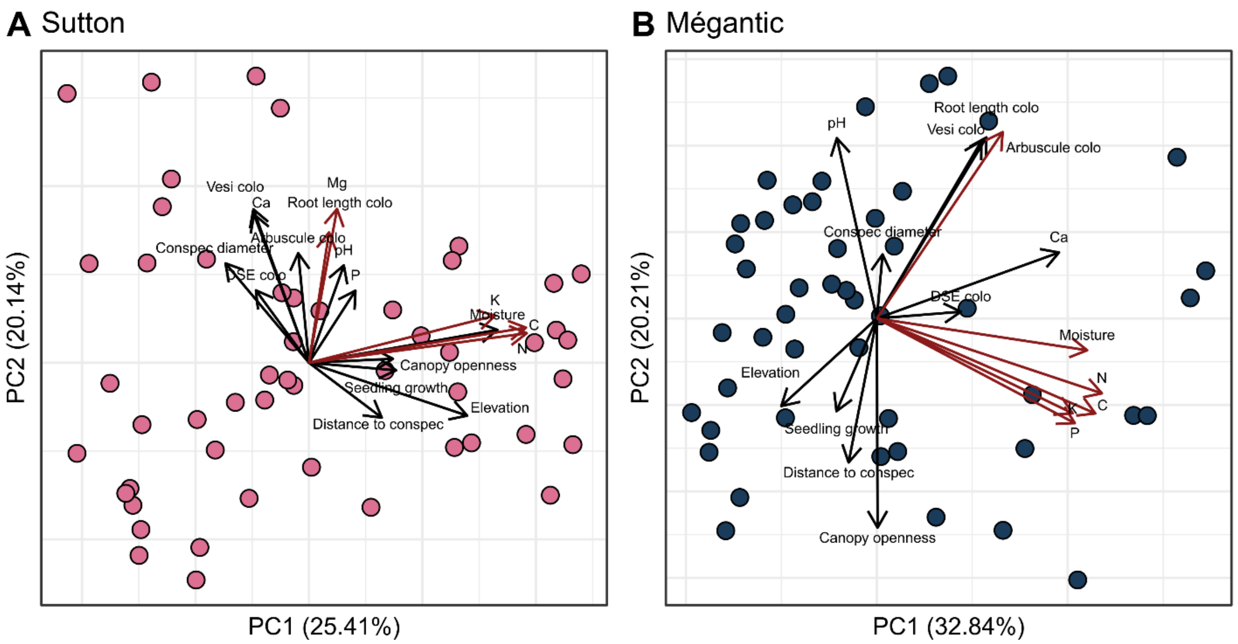


**Supplementary Figure 5.** PCA plots showing the multivariate relationships among abiotic and biotic predictors for Sutton (A) and Mégantic (B). The length and direction of the arrows represent the strength and orientation of associations of each predictor with the first two principal components. Notably, the red arrows show collinear predictors, which strongly correlate with other variables in the dataset. Due to their collinearity, these predictors were excluded when modeling linear (linear models) and multivariate relationships (PERMANOVA). The variance inflation factors (VIFs) were all lower than the recommended cut-off value of 3 in all final linear models, indicating that multicollinearity did not significantly affect model inference.

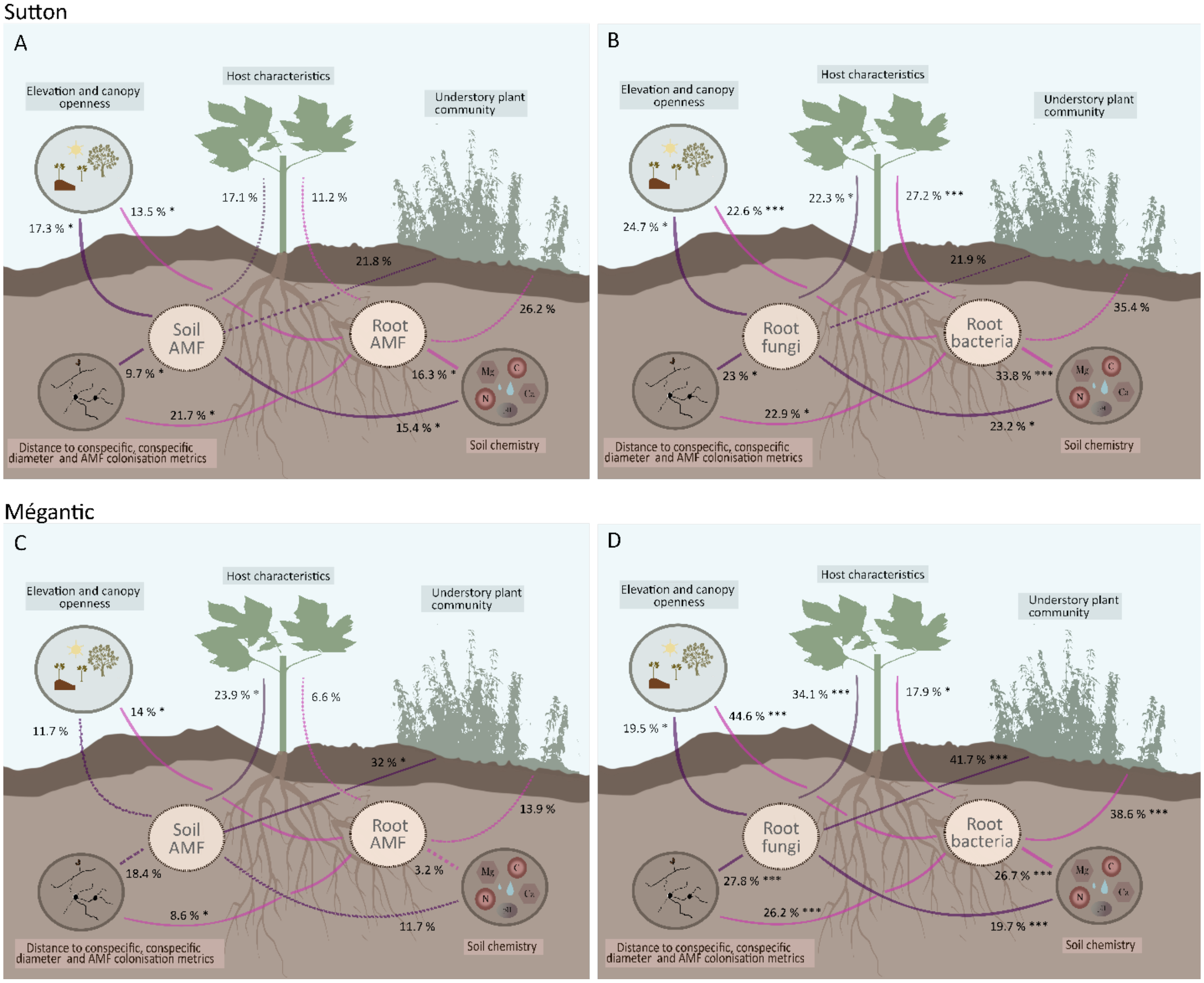


**Supplementary Figure 6.** Multivariate covariations between root microbial community matrices and (i) elevation and canopy openness, (ii) host characteristics, (iii) neighbouring plant community, (iv) distance to conspecific, conspecific diameter, and root microscopy and (v) soil chemistry.

(A and C) Multivariate covariations for root and soil AMF. (B and D) Multivariate covariations for fungal and bacterial communities. Line type indicates statistical significance (full lines) and insignificance (dashed). Numbers show RV coefficients (the degree of association between matrices in percentages). *$p \leq 0.05$, **$p \leq 0.01$, ***$p \leq 0.001$.

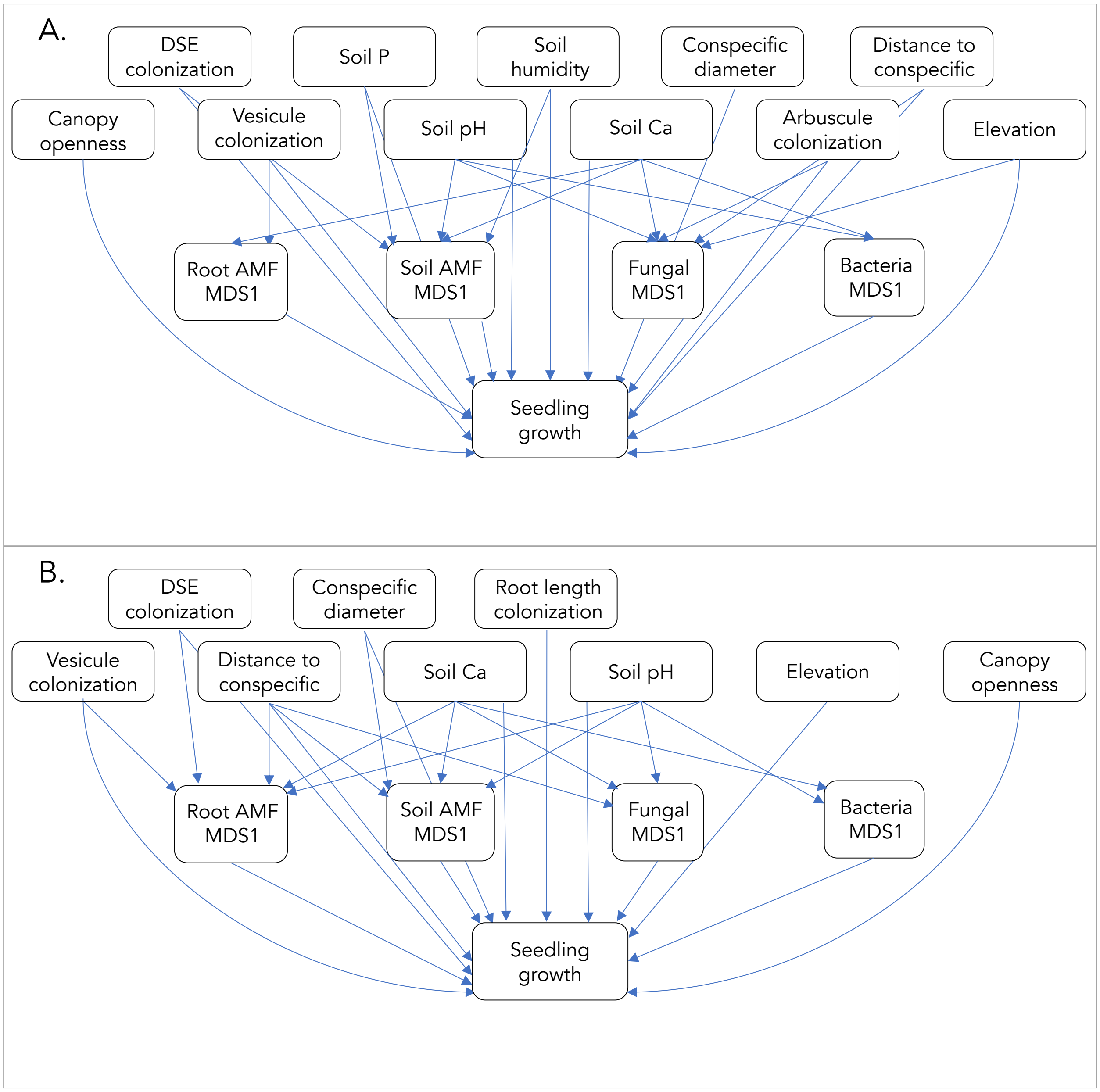


**Supplementary Figure 7**. Hypothetical structural equation models (SEM) representing the tested relationships among environmental factors, root microbial communities, and seedling growth. Panels show site identity (A: Sutton; B: Mégantic).

## REFERENCES


1. Chelius, M. K. & Triplett, E. W. The Diversity of Archaea and Bacteria in Association with the Roots of Zea mays L. *Microb Ecol* **41**, 252–263 (2001).
2. Gardes, M. & Bruns, T. D. ITS primers with enhanced specificity for basidiomycetes - application to the identification of mycorrhizae and rusts. *Molecular Ecology* **2**, 113–118 (1993).
3. White, T. J., Bruns, T., Lee, S. & Taylor, J. Amplification and direct sequencing of fungal ribosomal RNA genes for phylogenetics. in *PCR Protocols* (eds. White, T. J., Innis, M. A., Gelfand, D. H. & Sninsky, J. J.) 315–322 (Elsevier, New York, USA, 1990). doi:10.1016/B978-0-12-372180-8.50042-1.
4. Kolaříková, Z., Slavíková, R., Krüger, C., Krüger, M. & Kohout, P. PacBio sequencing of Glomeromycota rDNA: a novel amplicon covering all widely used ribosomal barcoding regions and its applicability in taxonomy and ecology of arbuscular mycorrhizal fungi. *New Phytologist* **231**, 490–499 (2021).
