# Supplementary material for: Interplay of biotic and abiotic factors shapes tree seedling growth and root-associated microbial communities
Source: Commun Biol. 2024 Mar 22;7:360. doi: 10.1038/s42003-024-06042-7 (PMC10960049; doi:10.1038/s42003-024-06042-7)
Supplement: Supplementary file 3 — Description of Additional Supplementary Files [file 42003_2024_6042_MOESM3_ESM.pdf]

Description of Additional Supplementary Files

File name: Supplementary Data 1

Description: Relationships between abiotic and biotic factors and root and soil AMF richness, diversity, and ordination scores (MDS1 and MDS2) at Sutton. Shown are t-values, *p*-values, standardized regression coefficients and its associated standard errors (SE), and adjusted coefficient of determination (R^2). Significant effects ($p \leq 0.05$) are in bold. MDS stands for Multi-Dimensional Scaling.

File name: Supplementary Data 2

Description: Relationships between abiotic and biotic factors and root and soil AMF richness, diversity, and ordination scores (MDS1 and MDS2) at Mégantic. Shown are t-values, *p*-values, standardized regression coefficients and its associated standard errors (SE), and adjusted coefficient of determination (R^2) for final models. Significant effects ($p \leq 0.05$) are in bold. MDS stands for Multi-Dimensional Scaling.

File name: Supplementary Data 3

Description: Relationships between abiotic and biotic factors with fungal and bacterial richness, diversity, and ordination scores (MDS1 and MDS2) at Sutton. Shown are t-values, *p*-values, standardized regression coefficients and its associated standard errors (SE), and adjusted coefficient of determination (R^2) for final models. Significant effects ($p \leq 0.05$) are in bold. MDS stands for Multi-Dimensional Scaling.

File name: Supplementary Data 4

Description: The relationship between abiotic and biotic factors and fungal and bacterial richness, diversity, and ordination scores (MDS1 and MDS2) at Mégantic. Shown are t-values, *p*-values, standardized regression coefficients and its associated standard errors (SE), and adjusted coefficient of determination (R^2) for final models. Significant effects ($p \leq 0.05$) are in bold. MDS stands for Multi-Dimensional Scaling.

File name: Supplementary Data 5

Description: The effect of abiotic and biotic factors on community composition of soil and root AMF, fungal and bacterial communities of Sugar maple. Shown are results from PERMANOVA on Bray-Curtis dissimilarity. Models were tested with *adonis2* function from the *vegan* package

with the argument *by=margin*. Partial coefficients of determination (R^2), sums of squares (SS) and associated significance levels (*p*-values) are demonstrated. Significant values ($p \leq 0.05$) are shown in bold.

File name: Supplementary Data 6

Description: Root fungal ASVs, taxonomy and functional guild assignments for Sutton.

File name: Supplementary Data 7

Description: Root fungal ASVs, taxonomy and functional guild assignments for Mégantic.
