# Supplementary material for: Interplay of biotic and abiotic factors shapes tree seedling growth and root-associated microbial communities
Source: Commun Biol. 2024 Mar 22;7:360. doi: 10.1038/s42003-024-06042-7 (PMC10960049; doi:10.1038/s42003-024-06042-7)
Supplement: Supplementary file 11 — Reporting Summary [file 42003_2024_6042_MOESM11_ESM.pdf]

nature portfolio

Corresponding author(s): Laforest-Lapointe, Isabelle; Faticov, Maria

Last updated by author(s): 2024/02/22

Reporting Summary

Nature Portfolio wishes to improve the reproducibility of the work that we publish. This form provides structure for consistency and transparency in reporting. For further information on Nature Portfolio policies, see our Editorial Policies and the Editorial Policy Checklist.

Statistics

For all statistical analyses, confirm that the following items are present in the figure legend, table legend, main text, or Methods section.

n/a	Confirmed	
☐	☒	The exact sample size (*n*) for each experimental group/condition, given as a discrete number and unit of measurement
☐	☒	A statement on whether measurements were taken from distinct samples or whether the same sample was measured repeatedly
☐	☒	The statistical test(s) used AND whether they are one- or two-sided<br>*Only common tests should be described solely by name; describe more complex techniques in the Methods section.*
☐	☒	A description of all covariates tested
☐	☒	A description of any assumptions or corrections, such as tests of normality and adjustment for multiple comparisons
☐	☒	A full description of the statistical parameters including central tendency (e.g. means) or other basic estimates (e.g. regression coefficient) AND variation (e.g. standard deviation) or associated estimates of uncertainty (e.g. confidence intervals)
☐	☒	For null hypothesis testing, the test statistic (e.g. *F*, *t*, *r*) with confidence intervals, effect sizes, degrees of freedom and *P* value noted<br>*Give P values as exact values whenever suitable.*
☒	☐	For Bayesian analysis, information on the choice of priors and Markov chain Monte Carlo settings
☐	☒	For hierarchical and complex designs, identification of the appropriate level for tests and full reporting of outcomes
☐	☒	Estimates of effect sizes (e.g. Cohen's *d*, Pearson's *r*), indicating how they were calculated

Our web collection on statistics for biologists contains articles on many of the points above.

Software and code

Policy information about availability of computer code

Data collection	No software was used to collect data in this study.
Data analysis	All metadata, taxa, and ASV tables are available on Figshare (10.6084/m9.figshare.25103738). All data analyses were conducted and visualizations generated in R (v. 4.2.1).

For manuscripts utilizing custom algorithms or software that are central to the research but not yet described in published literature, software must be made available to editors and reviewers. We strongly encourage code deposition in a community repository (e.g. GitHub). See the Nature Portfolio guidelines for submitting code & software for further information.

Data

Policy information about availability of data

All manuscripts must include a data availability statement. This statement should provide the following information, where applicable:

- Accession codes, unique identifiers, or web links for publicly available datasets
- A description of any restrictions on data availability
- For clinical datasets or third party data, please ensure that the statement adheres to our policy

All amplicon sequencing data generated in this study is deposited on the National Center for Biotechnology Information's (NCBI) Sequence Read Archive under BioProject accession number PRJNA1065908. All scripts for data analysis are available on Figshare (10.6084/m9.figshare.25103522).

Research involving human participants, their data, or biological material

Policy information about studies with human participants or human data. See also policy information about sex, gender (identity/presentation), and sexual orientation and race, ethnicity and racism.

Reporting on sex and gender	NA.
Reporting on race, ethnicity, or other socially relevant groupings	NA.
Population characteristics	NA.
Recruitment	NA.
Ethics oversight	NA.

Note that full information on the approval of the study protocol must also be provided in the manuscript.

Field-specific reporting

Please select the one below that is the best fit for your research. If you are not sure, read the appropriate sections before making your selection.

☐ Life sciences ☐ Behavioural & social sciences ☒ Ecological, evolutionary & environmental sciences

For a reference copy of the document with all sections, see nature.com/documents/nr-reporting-summary-flat.pdf

Ecological, evolutionary & environmental sciences study design

All studies must disclose on these points even when the disclosure is negative.

Study description	In this study, we investigated the drivers of tree root microbial endophyte communities (soil and root mycorrhizae, bacteria, and fungi) of sugar maple seedlings and assessed their relationship with seedling growth. We predicted that (1) local soil chemistry and neighbouring plant communities would be the main drivers of sugar maple root endophyte richness, diversity, and community composition; (2) seedling microbial richness, diversity, and community composition would shift across microbial kingdoms and elevation; (3) seedling growth would decrease with elevation because of shifts in microbial abundance and community composition (i.e., due to lower colonization by AMF at higher elevation or loss of symbionts) and unfavorable soil conditions (i.e., low pH and C, N, P, Mg, Ca, and K availability) closer to conifer-dense stands.<br>For each seedling, we measured mean annual tip growth (terminal internode length mm), age (years), height (cm), and root collar diameter (mm) as well as canopy openness (using 360° photos above each seedling using a Gap light analyzer). To capture understory plant communities in early season, for each plant species within 1m radius of each seedling we estimated percent cover using the following five classes: 1) 0% - understory plants are absent; 2) 1 - 25%, 3) 25% - 50%, 4) 50% - 75%, and 5) 75% - 100%. We also measured the distance to the closest adult conspecific tree (DBH >10cm) and its diameter at breast height. We collected seedling leaves, roots, and surrounding soil for chemical and molecular analyses. Leaf samples were used to measure foliar elemental concentrations (K, P, Ca, and Mg). Soil samples (20cm-deep cores, matching depth of root systems of seedlings) were used to characterize AMF communities and estimate soil properties (see below), while root samples were used to characterize endophytic AMF, bacteria, and fungi (see details below).
Research sample	Our study focuses on sugar maple seedling growth as well as root endophytic fungi, bacteria, and AMF along two elevational gradients located at Mont Écho near Sutton (45°6′46.09″N et 72°32′28.67″W; 811m) and Mont Saint-Joseph in Mont Mégantic National Park (45°26′51′′N, 71°06′52′′W; 1075m) in southern Québec, Canada.
Sampling strategy	50 seedlings per site were randomly selected to represent the full range of distribution of sugar maple along the two elevational gradients during exploratory visits by J. Chamard and their team. Preliminary visits allowed the team to identify starting points and the edge of the distribution of sugar maple at each site.
Data collection	Data and samples were collected during visits on sites in May and June 2021 by a team of researcher led by J. Chamard.
Timing and spatial scale	Data for local plant vegetation was collected in late May 2021 across two-day visits at each site. Seedlings and soils were collected in late June 2021 again across two-day visits at both sites. The gradient in Sutton occurs from 567 to 725 meters above sea level (m.a.s.l.) and from 675 to 796 meters m.a.s.l. in Mégantic. The two sites are separated by ~150km.
Data exclusions	Three seedlings were lost at Mégantic due to herbivory damages.
Reproducibility	Our findings are reproducible using the code provided in Figshare (10.6084/m9.figshare.25103522).
Randomization	Randomization was not relevant to our study as we aimed to compare the processes observed along the elevation gradients and across the sites.

Blinding	Blinding was not relevant to this study (see randomization).

Did the study involve field work? ☒ Yes ☐ No

Field work, collection and transport

Field conditions	Field work occurred in May and June of 2021. In late May, J. Chamard and their team flagged the seedlings and performed plant surveys at each plot. In late June, J. Chamard and their team collected the soil and plant samples. From 1981 to 2010, the mean annual temperature was 6.1ºC (value measured ~238 masl) and 4.0ºC (value measured ~240 masl), and the mean annual total precipitation was ~1310 mm and ~1370 mm, measured at the nearest weather station for Sutton and Mégantic, respectively.
Location	Sampling was conducted along two elevational gradients located at Mont Écho near Sutton (45°6′46.09″N et 72°32′28.67″W; 811m) and Mont Saint-Joseph in Mont Mégantic National Park (45°26′51′′N, 71°06′52′′W; 1075m) in southern Québec, Canada.
Access & import/export	Two permits were obtained to access and sample the sites: (1) for Mégantic, Mélina Dubois Verret from SEPAQ issued a special authorization for our work (PNMM-2021-05); and (2) for Sutton we obtained a permit (no permit number) from Mélanie Lelièvre/ David Brisson of Corridor Appalachien to access the permanent plot and acquire our samples.
Disturbance	Soil and seedling sampling (one sample of each per plot) caused minimal damage to the local vegetation. Access to the site was performed by walking without disturbing the forest stands.

Reporting for specific materials, systems and methods

We require information from authors about some types of materials, experimental systems and methods used in many studies. Here, indicate whether each material, system or method listed is relevant to your study. If you are not sure if a list item applies to your research, read the appropriate section before selecting a response.

Materials & experimental systems

n/a	Involved in the study	
☒	☐	Antibodies
☒	☐	Eukaryotic cell lines
☒	☐	Palaeontology and archaeology
☒	☐	Animals and other organisms
☒	☐	Clinical data
☒	☐	Dual use research of concern
☐	☒	Plants

Methods

n/a	Involved in the study	
☒	☐	ChIP-seq
☒	☐	Flow cytometry
☒	☐	MRI-based neuroimaging

Dual use research of concern

Policy information about dual use research of concern

Hazards

Could the accidental, deliberate or reckless misuse of agents or technologies generated in the work, or the application of information presented in the manuscript, pose a threat to:

No	Yes	
☒	☐	Public health
☒	☐	National security
☒	☐	Crops and/or livestock
☒	☐	Ecosystems
☒	☐	Any other significant area

Experiments of concern

Does the work involve any of these experiments of concern:

No	Yes	
☒	☐	Demonstrate how to render a vaccine ineffective
☒	☐	Confer resistance to therapeutically useful antibiotics or antiviral agents
☒	☐	Enhance the virulence of a pathogen or render a nonpathogen virulent
☒	☐	Increase transmissibility of a pathogen
☒	☐	Alter the host range of a pathogen
☒	☐	Enable evasion of diagnostic/detection modalities
☒	☐	Enable the weaponization of a biological agent or toxin
☒	☐	Any other potentially harmful combination of experiments and agents

Plants

Seed stocks	100 sugar maple seedlings were collected from two elevational gradients located at Mont Écho near Sutton (45°6′46.09″N et 72°32′ 28.67″W; 811m) and Mont Saint-Joseph in Mont Mégantic National Park (45°26′51′′N, 71°06′52′′W; 1075m) in southern Québec, Canada during the month of June, 2021.
Novel plant genotypes	NA.
Authentication	NA.
